# The Relationship between Symptom Flare of Atopic Dermatitis and Airborne Japanese Cedar and Cypress Pollen Counts: A Self-Scoring Diary Study

**DOI:** 10.5402/2012/218538

**Published:** 2012-04-09

**Authors:** Haruko Nishie, Mariko Kato, Shiori Kato, Hiroshi Odajima, Rumiko Shibata, Sankei Nishima, Reiko Kishikawa, Eiko Koto, Masutaka Furue

**Affiliations:** ^1^Department of Dermatology, Fukuoka National Hospital, National Hospital Organization, 4-39-1 Yakatabaru, Minami-ku, Fukuoka 811-1394, Japan; ^2^Department of Pediatrics, Fukuoka National Hospital, National Hospital Organization, Fukuoka 811-1394, Japan; ^3^Department of Allergology, Fukuoka National Hospital, National Hospital Organization, Fukuoka 811-1394, Japan; ^4^Kyushu Branch of Japan Allergy Foundation, Fukuoka 811-1394, Japan; ^5^Department of Dermatology, Graduate School of Medical Sciences, Kyushu University, Fukuoka 812-8582, Japan

## Abstract

*Background*. With an increase in Japanese cedar and cypress (JC) pollinosis, the relationship between JC pollen and atopic dermatitis (AD) has been studied. Some reports suggest that JC pollen can be one exacerbating factor for AD, but there has been no report that discusses JC pollen counts relating to AD symptom flare although actual airborne JC pollen counts can widely fluctuate throughout the pollen season. *Objective*. The relationship between symptom flare of AD and airborne JC pollen counts was examined. *Methods.* We monitored JC pollen counts in real time and divided the counts into low and high level. We then analyzed self-scored “itch intensity” recorded by 14 AD patients through a self-scoring diary. *Results*. Among the 14 patients, 7 had significantly higher itch intensity while the pollen counts were high. *Conclusion*. Even during the pollen season, actual airborne pollen counts can widely fluctuate. Our study suggested that symptom flare of AD could be influenced by the actual pollen counts.

## 1. Introduction

Japanese cedar and cypress (JC) pollinosis is a common disease in Japan. It is increasing dramatically [1, 2], and the sensitization rate among children also has been increasing [3, 4]. It is known that the cedar and cypress pollen grains have similar allergens. With increasing prevalence, various kinds of pollen have been focused on as potential triggers of dermatitis and other diseases such as oral allergy syndrome [2, 5–7].

Previous studies have concluded that pollen can effect atopic dermatitis (AD), reporting many AD patients showing symptom flare from February to May [8, 9], during which time the airborne JC pollen level is high in Japan. February to May is called “JC pollen season” in Japan, but actual airborne JC pollen counts can widely fluctuate throughout the season, which means variations in the level of exposure to pollen. To the best of our knowledge, there has been no report that discusses JC pollen counts relating to AD symptom flare. Thus, we monitored JC pollen counts in real time and examined the relationship between AD symptom flare and airborne JC pollen counts through an individual self-scoring diary.

## 2. Methods

### 2.1. Individual Self-Scoring Diary

We asked AD patients diagnosed according to Japanese Dermatological Association Criteria for the diagnosis of AD, who attended our hospitals between February 1 and March 31, 2007 or 2011, to self-score their itch intensity every day from the day they were given a self-scoring diary until May 31. If the patient was too young to self-score, his/her family was asked to record the itch intensity. The itch intensity was scored by visual analogue scale from 0 (no itch) to 100 (the strongest itch intensity they had ever experienced). Oral and topical treatments were continued throughout the study as they were prior to enrolling in this study. This study has been approved by the Ethics Committee of Fukuoka National Hospital, and all participants or their family have given their informed consent. We had 57 AD patients who agreed to join this study, but 43 patients dropped out. As a result, 14 patients (6 male; 8 female; ages from 2 to 53; mean age 27.0) completed the self-scoring diary.

### 2.2. Monitoring of JC Pollen Counts

We monitored airborne JC pollen counts of our hospitals area, which is a part of Fukuoka Prefecture, southern region in Japan, every day for 3 months (February 1 to April 30, 2007 and 2011) using a gravitational method (Durham's sampler). This method is simple and useful to collect data [10], and it is the most commonly used method in Japan. It has also been reported that JC pollen counts measured with a Durham's sampler and a Burkard sampler were correlative [11]. Because airborne pollen counts of 5/cm^2^ or more are considered high-exposure in Japan, we defined a period of days with the airborne pollen counts of 5/cm^2^ or more as a high-exposure period and a period of days with the airborne pollen counts of less than 5/cm^2^ as a low-exposure period. We then analyzed the self-scored itch intensity recorded by each patient during the high- and low-exposure periods. The patients were blinded from our pollen count information.

### 2.3. Statistical Analysis

We compared mean scores of high- and low-exposure periods. Statistical analysis was done using Student's *t*-test or Welch's *t*-test. A *P* value less than 0.05 was considered significant.

## 3. Results

**Figure 1:**
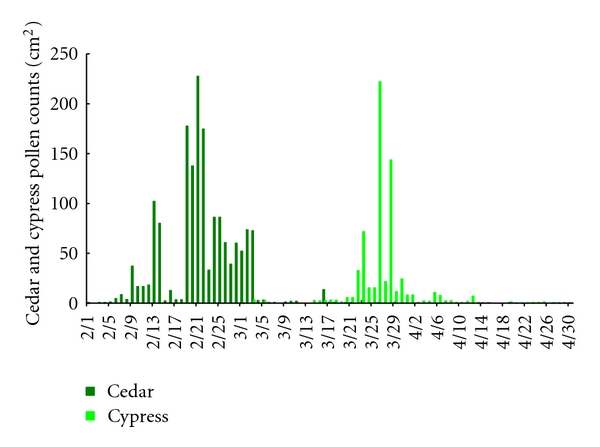
Pollen counts monitored with a Durham's sampler from February 1 to April 30, 2007.

**Figure 2:**
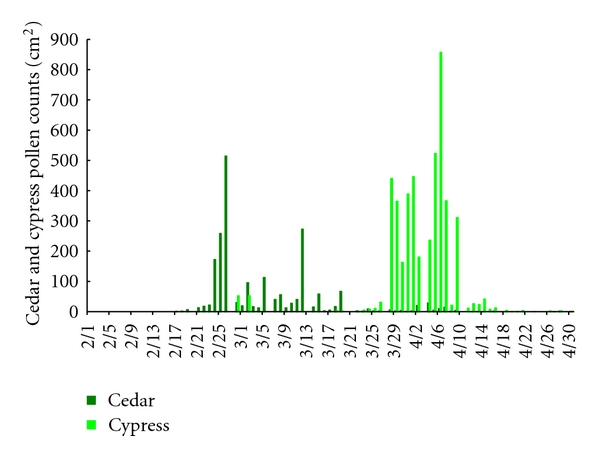
Pollen counts monitored with a Durham's sampler from February 1 to April 30, 2011.

The monitored pollen counts are shown in Figures 1 and 2. During the course of this study in 2007 (February 1 to April 30, 2007), February 9 to March 3 corresponded to a high-exposure period for cedar pollen, and March 20 to April 6 corresponded to a high-exposure period for cypress pollen. During the course of this study in 2011 (February 1 to April 30, 2011), February 19 to March 19 and April 2 to 7 corresponded to a high-exposure period for cedar pollen, and March 24 to April 16 corresponded to a high-exposure period for cypress pollen. All other times corresponded to a low-exposure period for both types of pollen.

**Figure 3:**
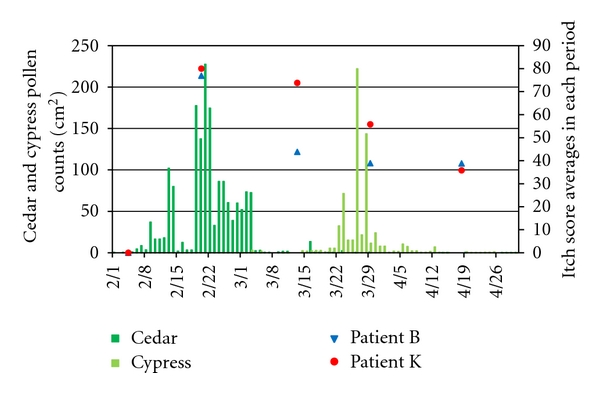
Itch score averages for Patients B and K in each period. Note: itch score averages changed along with the pollen counts of Japanese cedar.

We got 11 patients (Patients A–K) in 2007 and 3 patients (Patients L–N) in 2011, and the results are shown in Table 1 with patients' backgrounds. Among the 14 patients, 7 patients (Patients B, D, F, J, K, L, and N) reported significantly higher itch scores during the high-exposure period for cedar (Patients B, F, J, K, and N) or cypress (Patients D, L, and N) pollen compared to the low-exposure period. For Patients I and M, the itch scores significantly decreased during the high-exposure period compared to the low-exposure period for cypress (Patient I) or both (Patient M) pollen. Chronological itch scores for two representative patients (Patients B and K) were depicted with the cedar and cypress pollen counts in Figure 3. For these patients, itch scores rapidly increased along with the beginning of the high-exposure period for cedar pollen compared to the preseasonal period and gradually decreased after the cessation of cedar pollen scattering.

## 4. Discussion

Symptoms of AD are considered to be attributed to the complicated combination of a type I to IV allergy reaction [12, 13]. AD eczematous lesions are generally observed 6–24 hours after exposure to a specific allergen [14, 15], and it is suggested that IgE-mediated late phase reaction (LPR) plays a part in this phenomenon. Other than previous reports [8, 9], which showed that many AD patients had symptom flare during the pollen season, our study suggested that the symptom flare could be influenced by actual airborne JC pollen counts. Because barrier function of skin is damaged, AD patients can be sensitized more easily, and eczematous lesions can be quickly caused by attached allergens. This theory can explain the relationship between AD symptom flare and airborne pollen counts. On the other hand, there were two patients who had itch flare during the low-exposure period. Specific allergens responsible for their AD symptom flare could not be ascertained over the course of this study.

Some reports showed that there was no difference in specific IgE level and a positive skin prick test between two groups who had or did not have symptom flare during the pollen season [8, 9], although a positive patch test was significantly high among the patients who had symptom flare during the pollen season [9]. In addition, some reports have denied the correlation between specific IgE level and LPR to the same allergen [15–17]. In our study, 11 patients were positive specific IgE to Japanese cedar pollen (CAP-RAST class ≥2), and among them 3 patients had itch flare during the high-exposure period for cedar pollen, whereas 6 patients did not. The specific IgE levels did not influence the frequency of itch flare, and this result may be consistent with the suggestion of the previous reports. In addition, most patients did not have seasonal allergic rhinitis in JC pollen season. These may suggest that symptoms of AD cannot be attributed to type I allergy reaction but to mainly LPR or type IV allergy reaction. However, the detailed mechanisms involved in the exacerbation of AD during the pollen season are still unknown.

There are a few limitations in our study. First, itch is only one of several AD symptoms. Second, the itch intensity was not evaluated objectively by a physician. Oral and topical treatments were continued throughout this study as they were used prior to enrollment, which could have effects on itch scores. These limitations could not be analyzed.

In conclusion, JC pollen can be an exacerbating factor for AD, and it may make symptoms worse depending on airborne pollen counts. The frequency of symptom flare during the JC pollen season may not depend on IgE levels or the presence of seasonal allergic rhinitis.

## Figures and Tables

**Table 1:** Self-scored itch intensity during the high- and low-exposure periods and backgrounds for each patient.

Patient (age, gender)	During high-exposure period for cedar	During high-exposure period for cypress	During low-exposure period	Severity of AD	Total IgE (IU/mL)	Specific IgE to cedar pollen (CAP-RAST class)	Seasonal allergic rhinitis
A (11, M)	70.0 ± 0.0	70.0 ± 0.0	70.0 ± 0.0	mild	4680	2	−
B (2, M)	76.9 ± 19.6*	38.9 ± 20.3	40.8 ± 20.3	mild	550	no data	−
C (13, F)	75.8 ± 8.4	83.9 ± 9.9	82.9 ± 18.0	severe	35220	4	−
D (13, M)	62.5 ± 2.6	63.2 ± 2.5*	60.5 ± 2.8	moderate	5040	4	−
E (3, F)	22.8 ± 6.9	23.3 ± 3.8	24.4 ± 5.0	severe	23994	2	−
F (53, M)	29.1 ± 3.0*	24.4 ± 5.1	23.4 ± 4.8	mild	2436	4	+
G (35, F)	51.7 ± 20.7	60.8 ± 13.0	56.3 ± 9.0	mild	41	0	+
H (36, F)	no data	19.2 ± 16.7	13.0 ± 12.1	mild	1610	5	no data
I (31, F)	no data	10.0 ± 0.0**	33.0 ± 19.4	moderate	3336	2	no data
J (16, M)	68.2 ± 3.5*	62.9 ± 2.8	63.0 ± 5.2	moderate	2884	5	−
K (43, F)	80.0 ± 9.1*	55.8 ± 15.1	51.0 ± 21.1	moderate	85	2	−
L (43, F)	47.7 ± 12.8	55.7 ± 6.5*	49.6 ± 11.2	moderate	106	4	+
M (39, F)	63.3 ± 7.1**	58.3 ± 10.1**	70.6 ± 5.4	moderate	116	2	no data
N (40, M)	70.1 ± 9.0*	69.2 ± 7.5*	53.9 ± 12.3	moderate	no data	no data	+

Data are average ± standard deviation. *means significantly high and **means significantly low compared to low-exposure period.

Severity of AD was evaluated according to the classification by Japanese Ministry of Health, Labour and Welfare Study Group (mild: only slight eruptions, moderate: with severe eruptions on less than 10% of body, severe: with severe eruptions on 10% to less than 30% of body, very severe: with severe eruptions on 30% or more of body).
